# Supplementary material for: Variable Correlation between Bronchoalveolar Lavage Fluid Fungal Load and Serum-(1,3)-β-d-Glucan in Patients with Pneumocystosis—A Multicenter ECMM Excellence Center Study
Source: J Fungi (Basel). 2020 Dec 1;6(4):327. doi: 10.3390/jof6040327 (PMC7711754; doi:10.3390/jof6040327)
Supplement: Supplementary file 1 [file jof-06-00327-s001.pdf]

Journal of Fungi

MDPI

Supplementary Materials

Table S1.

	qPCR assay evaluation (PMID)	Manufacturer	Extraction method
Center 1	19846641	In house	Extraction from 1 mL of sample using NucliSens easyMAG/eMAG (BioMérieux, France)<br>Pretreatment and lysis in GuSCN-buffer<br>Binding to silica coated magnetic beads<br>Elution into final volume of 50 µL
Center 2	20946413	In house	No bead beating<br>Qiasymphony (Qiagen)<br>Virus Pathogen extraction kit (Qiagen)<br>Extracted volume (1mL)<br>Elution 85µL
Center 3	31758173	RidaGene	Extraction from 500 µl of BAL fluid using the NucliSENS® easyMAG® instrument (specific B protocol). The elution volume was 50 µl. Amplification and detection using the RIDA®GENE Pneumocystis jirovecii kit according to the manufacturer's instructions (r-Biopharm).
Center 4	30430780	Altona Diagnostics	No beat beating; pretreatment with Sputasol (Oxoid LTD, Hamshire, England), 300 ul sample; Extraction in Maxwell 16 instrument (Promega GmbH, Walldorf, Germany); Elution 50 ul
Center 5	1974987	Tibmolbiol	High Pure Viral Nucleic Acid Kit (Roche); extraction volume 200 µL

Table S2: ROC curve sensibility and specificity data for BAL qPCR.

	Sensitivity%	95% CI	Specificity%	95% CI	Likelihood ratio
> 17.57	100,0	88,65% to 100,0%	0,8547	0,04384% to 4,683%	1,009
> 19.46	100,0	88,65% to 100,0%	1,709	0,3037% to 6,019%	1,017
> 20.01	100,0	88,65% to 100,0%	2,564	0,6989% to 7,268%	1,026
> 20.10	100,0	88,65% to 100,0%	3,419	1,337% to 8,462%	1,035
> 20.45	100,0	88,65% to 100,0%	4,274	1,839% to 9,615%	1,045
> 20.87	100,0	88,65% to 100,0%	5,128	2,371% to 10,74%	1,054
> 21.38	100,0	88,65% to 100,0%	7,692	4,100% to 13,97%	1,083
> 21.79	100,0	88,65% to 100,0%	8,547	4,709% to 15,02%	1,093
> 21.89	100,0	88,65% to 100,0%	9,402	5,331% to 16,05%	1,104
> 22.00	100,0	88,65% to 100,0%	11,11	6,609% to 18,09%	1,125
> 22.12	100,0	88,65% to 100,0%	11,97	7,263% to 19,09%	1,136
> 22.27	100,0	88,65% to 100,0%	12,82	7,926% to 20,08%	1,147
> 22.62	100,0	88,65% to 100,0%	13,68	8,596% to 21,06%	1,158
> 22.95	100,0	88,65% to 100,0%	14,53	9,274% to 22,04%	1,170
> 23.15	100,0	88,65% to 100,0%	16,24	10,65% to 23,98%	1,194
> 23.65	100,0	88,65% to 100,0%	17,09	11,35% to 24,93%	1,206
> 24.10	100,0	88,65% to 100,0%	19,66	13,47% to 27,77%	1,245
> 24.23	100,0	88,65% to 100,0%	20,51	14,19% to 28,71%	1,258
> 24.46	100,0	88,65% to 100,0%	21,37	14,91% to 29,64%	1,272
> 24.83	100,0	88,65% to 100,0%	22,22	15,64% to 30,57%	1,286
> 25.37	100,0	88,65% to 100,0%	23,08	16,37% to 31,49%	1,300
> 25.80	100,0	88,65% to 100,0%	23,93	17,11% to 32,41%	1,315
> 25.90	100,0	88,65% to 100,0%	24,79	17,85% to 33,33%	1,330
> 25.99	100,0	88,65% to 100,0%	30,77	23,13% to 39,63%	1,444
> 26.02	100,0	88,65% to 100,0%	31,62	23,90% to 40,52%	1,463
> 26.08	100,0	88,65% to 100,0%	32,48	24,67% to 41,40%	1,481

> 26.54	100,0	88,65% to 100,0%	33,33	25,44% to 42,28%	1,500
> 26.96	100,0	88,65% to 100,0%	34,19	26,22% to 43,16%	1,519
> 26.97	100,0	88,65% to 100,0%	35,04	27,00% to 44,04%	1,539
> 26.98	100,0	88,65% to 100,0%	38,46	30,15% to 47,51%	1,625
> 27.06	100,0	88,65% to 100,0%	39,32	30,94% to 48,37%	1,648
> 27.26	100,0	88,65% to 100,0%	40,17	31,74% to 49,23%	1,671
> 27.46	100,0	88,65% to 100,0%	41,03	32,54% to 50,09%	1,696
> 27.61	100,0	88,65% to 100,0%	41,88	33,34% to 50,94%	1,721
> 27.72	96,67	83,33% to 99,83%	41,88	33,34% to 50,94%	1,663
> 27.83	96,67	83,33% to 99,83%	42,74	34,14% to 51,79%	1,688
> 27.93	96,67	83,33% to 99,83%	43,59	34,95% to 52,64%	1,714
> 27.97	96,67	83,33% to 99,83%	44,44	35,76% to 53,48%	1,740
> 28.04	96,67	83,33% to 99,83%	52,14	43,16% to 60,98%	2,020
> 28.19	96,67	83,33% to 99,83%	52,99	44,00% to 61,80%	2,056
> 28.38	96,67	83,33% to 99,83%	53,85	44,83% to 62,61%	2,094
> 28.49	96,67	83,33% to 99,83%	54,70	45,67% to 63,43%	2,134
> 28.69	93,33	78,68% to 98,82%	54,70	45,67% to 63,43%	2,060
> 28.93	93,33	78,68% to 98,82%	55,56	46,52% to 64,24%	2,100
> 29.02	93,33	78,68% to 98,82%	59,83	50,77% to 68,26%	2,323
> 29.11	93,33	78,68% to 98,82%	60,68	51,63% to 69,06%	2,374
> 29.22	93,33	78,68% to 98,82%	61,54	52,49% to 69,85%	2,427
> 29.37	90,00	74,38% to 96,54%	61,54	52,49% to 69,85%	2,340
> 29.64	90,00	74,38% to 96,54%	62,39	53,35% to 70,64%	2,393
> 29.89	90,00	74,38% to 96,54%	63,25	54,22% to 71,43%	2,449
> 29.97	90,00	74,38% to 96,54%	64,10	55,09% to 72,22%	2,507
> 30.00	90,00	74,38% to 96,54%	66,67	57,72% to 74,56%	2,700
> 30.10	90,00	74,38% to 96,54%	67,52	58,60% to 75,33%	2,771
> 30.21	90,00	74,38% to 96,54%	68,38	59,48% to 76,10%	2,846
> 30.26	90,00	74,38% to 96,54%	69,23	60,37% to 76,87%	2,925
> 30.43	90,00	74,38% to 96,54%	70,09	61,26% to 77,64%	3,009
> 30.60	90,00	74,38% to 96,54%	70,94	62,15% to 78,40%	3,097
> 30.70	90,00	74,38% to 96,54%	71,79	63,05% to 79,16%	3,191
> 30.82	90,00	74,38% to 96,54%	72,65	63,95% to 79,91%	3,291
> 30.87	90,00	74,38% to 96,54%	74,36	65,76% to 81,41%	3,510
> 30.89	90,00	74,38% to 96,54%	76,07	67,59% to 82,89%	3,761
> 30.99	90,00	74,38% to 96,54%	76,92	68,51% to 83,63%	3,900
> 31.18	90,00	74,38% to 96,54%	77,78	69,43% to 84,36%	4,050
> 31.28	90,00	74,38% to 96,54%	78,63	70,36% to 85,09%	4,212
> 31.29	86,67	70,32% to 94,69%	78,63	70,36% to 85,09%	4,056
> 31.39	86,67	70,32% to 94,69%	79,49	71,29% to 85,81%	4,225
> 31.53	86,67	70,32% to 94,69%	80,34	72,23% to 86,53%	4,409
> 31.73	86,67	70,32% to 94,69%	81,20	73,17% to 87,24%	4,609
> 31.93	86,67	70,32% to 94,69%	82,05	74,11% to 87,95%	4,829
> 32.13	86,67	70,32% to 94,69%	82,91	75,07% to 88,65%	5,070
> 32.28	86,67	70,32% to 94,69%	83,76	76,02% to 89,35%	5,337
> 32.38	86,67	70,32% to 94,69%	84,62	76,99% to 90,04%	5,633
> 32.48	86,67	70,32% to 94,69%	85,47	77,96% to 90,73%	5,965
> 32.53	86,67	70,32% to 94,69%	87,18	79,92% to 92,07%	6,760
> 32.59	86,67	70,32% to 94,69%	88,03	80,91% to 92,74%	7,243
> 32.75	86,67	70,32% to 94,69%	88,89	81,91% to 93,39%	7,800
> 32.94	83,33	66,44% to 92,66%	88,89	81,91% to 93,39%	7,500
> 33.03	83,33	66,44% to 92,66%	90,60	83,95% to 94,67%	8,864
> 33.13	83,33	66,44% to 92,66%	91,45	84,98% to 95,29%	9,750
> 33.30	80,00	62,69% to 90,49%	91,45	84,98% to 95,29%	9,360
> 33.46	76,67	59,07% to 88,21%	91,45	84,98% to 95,29%	8,970
> 33.54	73,33	55,55% to 85,82%	91,45	84,98% to 95,29%	8,580

> 33.63	73,33	55,55% to 85,82%	92,31	86,03% to 95,90%	9,533
> 33.73	73,33	55,55% to 85,82%	93,16	87,09% to 96,49%	10,73
> 33.85	70,00	52,12% to 83,34%	93,16	87,09% to 96,49%	10,24
> 33.95	70,00	52,12% to 83,34%	94,02	88,16% to 97,07%	11,70
> 33.99	70,00	52,12% to 83,34%	95,73	90,38% to 98,16%	16,38
> 34.08	66,67	48,78% to 80,77%	95,73	90,38% to 98,16%	15,60
> 34.17	63,33	45,51% to 78,13%	95,73	90,38% to 98,16%	14,82
> 34.28	63,33	45,51% to 78,13%	96,58	91,54% to 98,66%	18,53
> 34.46	60,00	42,32% to 75,41%	96,58	91,54% to 98,66%	17,55
> 34.55	56,67	39,20% to 72,62%	96,58	91,54% to 98,66%	16,58
> 34.67	53,33	36,14% to 69,77%	96,58	91,54% to 98,66%	15,60
> 34.82	53,33	36,14% to 69,77%	97,44	92,73% to 99,30%	20,80
> 34.96	50,00	33,15% to 66,85%	97,44	92,73% to 99,30%	19,50
> 35.09	50,00	33,15% to 66,85%	98,29	93,98% to 99,70%	29,25
> 35.14	46,67	30,23% to 63,86%	98,29	93,98% to 99,70%	27,30
> 35.49	43,33	27,38% to 60,80%	98,29	93,98% to 99,70%	25,35
> 35.85	40,00	24,59% to 57,68%	98,29	93,98% to 99,70%	23,40
> 35.89	40,00	24,59% to 57,68%	99,15	95,32% to 99,96%	46,80
> 35.94	36,67	21,87% to 54,49%	99,15	95,32% to 99,96%	42,90
> 36.03	33,33	19,23% to 51,22%	99,15	95,32% to 99,96%	39,00
> 36.14	33,33	19,23% to 51,22%	100,0	96,82% to 100,0%	
> 36.29	30,00	16,66% to 47,88%	100,0	96,82% to 100,0%	
> 36.58	26,67	14,18% to 44,45%	100,0	96,82% to 100,0%	
> 36.82	23,33	11,79% to 40,93%	100,0	96,82% to 100,0%	
> 37.06	20,00	9,505% to 37,31%	100,0	96,82% to 100,0%	
> 37.34	16,67	7,337% to 33,56%	100,0	96,82% to 100,0%	
> 37.46	13,33	5,310% to 29,68%	100,0	96,82% to 100,0%	
> 37.78	10,00	3,460% to 25,62%	100,0	96,82% to 100,0%	
> 38.19	6,667	1,185% to 21,32%	100,0	96,82% to 100,0%	
> 38.34	3,333	0,1710% to 16,67%	100,0	96,82% to 100,0%	

Table S3: ROC curve sensibility and specificity data for BDG.

	Sensitivity%	95% CI	Specificity%	95% CI	Likelihood ratio
< 8.350	26,67	14,18% to 44,45%	100,0	96,82% to 100,0%	
< 9.070	30,00	16,66% to 47,88%	100,0	96,82% to 100,0%	
< 9.365	33,33	19,23% to 51,22%	100,0	96,82% to 100,0%	
< 10.53	36,67	21,87% to 54,49%	100,0	96,82% to 100,0%	
< 12.23	40,00	24,59% to 57,68%	100,0	96,82% to 100,0%	
< 13.50	46,67	30,23% to 63,86%	100,0	96,82% to 100,0%	
< 14.70	46,67	30,23% to 63,86%	99,15	95,32% to 99,96%	54,60
< 16.20	50,00	33,15% to 66,85%	95,73	90,38% to 98,16%	11,70
< 17.10	50,00	33,15% to 66,85%	94,87	89,26% to 97,63%	9,750
< 17.70	53,33	36,14% to 69,77%	94,87	89,26% to 97,63%	10,40
< 19.60	56,67	39,20% to 72,62%	94,87	89,26% to 97,63%	11,05
< 23.75	60,00	42,32% to 75,41%	94,87	89,26% to 97,63%	11,70
< 27.75	60,00	42,32% to 75,41%	94,02	88,16% to 97,07%	10,03
< 29.24	63,33	45,51% to 78,13%	94,02	88,16% to 97,07%	10,59
< 29.74	66,67	48,78% to 80,77%	94,02	88,16% to 97,07%	11,14
< 30.50	70,00	52,12% to 83,34%	94,02	88,16% to 97,07%	11,70
< 31.15	70,00	52,12% to 83,34%	87,18	79,92% to 92,07%	5,460
< 34.48	73,33	55,55% to 85,82%	87,18	79,92% to 92,07%	5,720
< 38.96	73,33	55,55% to 85,82%	86,32	78,94% to 91,40%	5,363
< 41.10	73,33	55,55% to 85,82%	85,47	77,96% to 90,73%	5,047
< 42.60	73,33	55,55% to 85,82%	84,62	76,99% to 90,04%	4,767
< 49.86	73,33	55,55% to 85,82%	83,76	76,02% to 89,35%	4,516
< 64.49	73,33	55,55% to 85,82%	82,91	75,07% to 88,65%	4,290
< 74.77	73,33	55,55% to 85,82%	82,05	74,11% to 87,95%	4,086
< 78.42	76,67	59,07% to 88,21%	82,05	74,11% to 87,95%	4,271
< 82.92	76,67	59,07% to 88,21%	81,20	73,17% to 87,24%	4,077
< 89.00	80,00	62,69% to 90,49%	81,20	73,17% to 87,24%	4,255
< 92.16	83,33	66,44% to 92,66%	81,20	73,17% to 87,24%	4,432
< 92.66	83,33	66,44% to 92,66%	80,34	72,23% to 86,53%	4,239
< 93.70	83,33	66,44% to 92,66%	79,49	71,29% to 85,81%	4,063
< 96.00	86,67	70,32% to 94,69%	79,49	71,29% to 85,81%	4,225
< 97.66	90,00	74,38% to 96,54%	79,49	71,29% to 85,81%	4,388
< 100.2	90,00	74,38% to 96,54%	78,63	70,36% to 85,09%	4,212
< 109.7	90,00	74,38% to 96,54%	77,78	69,43% to 84,36%	4,050
< 128.3	93,33	78,68% to 98,82%	77,78	69,43% to 84,36%	4,200
< 144.1	96,67	83,33% to 99,83%	77,78	69,43% to 84,36%	4,350
< 148.6	96,67	83,33% to 99,83%	76,92	68,51% to 83,63%	4,189
< 150.7	96,67	83,33% to 99,83%	76,07	67,59% to 82,89%	4,039
< 158.0	96,67	83,33% to 99,83%	75,21	66,67% to 82,15%	3,900
< 173.8	96,67	83,33% to 99,83%	74,36	65,76% to 81,41%	3,770
< 185.2	96,67	83,33% to 99,83%	73,50	64,85% to 80,66%	3,648
< 188.8	96,67	83,33% to 99,83%	72,65	63,95% to 79,91%	3,534
< 191.2	100,0	88,65% to 100,0%	72,65	63,95% to 79,91%	3,656
< 191.5	100,0	88,65% to 100,0%	71,79	63,05% to 79,16%	3,545
< 197.2	100,0	88,65% to 100,0%	70,94	62,15% to 78,40%	3,441
< 205.3	100,0	88,65% to 100,0%	70,09	61,26% to 77,64%	3,343
< 212.9	100,0	88,65% to 100,0%	69,23	60,37% to 76,87%	3,250
< 222.0	100,0	88,65% to 100,0%	68,38	59,48% to 76,10%	3,162
< 228.6	100,0	88,65% to 100,0%	67,52	58,60% to 75,33%	3,079
< 234.7	100,0	88,65% to 100,0%	66,67	57,72% to 74,56%	3,000
< 240.1	100,0	88,65% to 100,0%	65,81	56,84% to 73,78%	2,925
< 242.8	100,0	88,65% to 100,0%	64,96	55,96% to 73,00%	2,854
< 245.2	100,0	88,65% to 100,0%	64,10	55,09% to 72,22%	2,786

< 262.3	100,0	88,65% to 100,0%	63,25	54,22% to 71,43%	2,721
< 279.0	100,0	88,65% to 100,0%	62,39	53,35% to 70,64%	2,659
< 298.1	100,0	88,65% to 100,0%	61,54	52,49% to 69,85%	2,600
< 321.9	100,0	88,65% to 100,0%	60,68	51,63% to 69,06%	2,543
< 328.9	100,0	88,65% to 100,0%	59,83	50,77% to 68,26%	2,489
< 331.0	100,0	88,65% to 100,0%	58,97	49,91% to 67,46%	2,438
< 334.4	100,0	88,65% to 100,0%	58,12	49,06% to 66,66%	2,388
< 343.8	100,0	88,65% to 100,0%	57,26	48,21% to 65,86%	2,340
< 350.9	100,0	88,65% to 100,0%	56,41	47,36% to 65,05%	2,294
< 360.5	100,0	88,65% to 100,0%	55,56	46,52% to 64,24%	2,250
< 376.7	100,0	88,65% to 100,0%	54,70	45,67% to 63,43%	2,208
< 386.7	100,0	88,65% to 100,0%	53,85	44,83% to 62,61%	2,167
< 401.0	100,0	88,65% to 100,0%	52,99	44,00% to 61,80%	2,127
< 413.8	100,0	88,65% to 100,0%	52,14	43,16% to 60,98%	2,089
< 427.0	100,0	88,65% to 100,0%	51,28	42,33% to 60,15%	2,053
< 445.2	100,0	88,65% to 100,0%	50,43	41,50% to 59,33%	2,017
< 464.2	100,0	88,65% to 100,0%	49,57	40,67% to 58,50%	1,983
< 481.9	100,0	88,65% to 100,0%	48,72	39,85% to 57,67%	1,950
< 489.2	100,0	88,65% to 100,0%	47,86	39,02% to 56,84%	1,918
< 495.5	100,0	88,65% to 100,0%	47,01	38,20% to 56,00%	1,887
< 507.2	100,0	88,65% to 100,0%	41,88	33,34% to 50,94%	1,721
< 517.2	100,0	88,65% to 100,0%	41,03	32,54% to 50,09%	1,696
< 522.0	100,0	88,65% to 100,0%	40,17	31,74% to 49,23%	1,671
